# AUG_hairpin: prediction of a downstream secondary structure influencing the recognition of a translation start site

**DOI:** 10.1186/1471-2105-8-318

**Published:** 2007-08-30

**Authors:** Alex V Kochetov, Andrey Palyanov, Igor I Titov, Dmitry Grigorovich, Akinori Sarai, Nikolay A Kolchanov

**Affiliations:** 1 Institute of Cytology and Genetics, Lavrentieva 10, Novosibirsk 630090, Russia; 2 Novosibirsk State University, Novosibirsk 630090, Russia; 3 Kyushu Institute of Technology, Iizuka, 820-8502, Japan

## Abstract

**Background:**

The translation start site plays an important role in the control of translation efficiency of eukaryotic mRNAs. The recognition of the start AUG codon by eukaryotic ribosomes is considered to depend on its nucleotide context. However, the fraction of eukaryotic mRNAs with the start codon in a suboptimal context is relatively large. It may be expected that mRNA should possess some features providing efficient translation, including the proper recognition of a translation start site. It has been experimentally shown that a downstream hairpin located in certain positions with respect to start codon can compensate in part for the suboptimal AUG context and also increases translation from non-AUG initiation codons. Prediction of such a compensatory hairpin may be useful in the evaluation of eukaryotic mRNA translation properties.

**Results:**

We evaluated interdependency between the start codon context and mRNA secondary structure at the CDS beginning: it was found that a suboptimal start codon context significantly correlated with higher base pairing probabilities at positions 13 – 17 of CDS of human and mouse mRNAs. It is likely that the downstream hairpins are used to enhance translation of some mammalian mRNAs *in vivo*. Thus, we have developed a tool, *AUG_hairpin*, to predict local stem-loop structures located within the defined region at the beginning of mRNA coding part. The implemented algorithm is based on the available published experimental data on the CDS-located stem-loop structures influencing the recognition of upstream start codons.

**Conclusion:**

An occurrence of a potential secondary structure downstream of start AUG codon in a suboptimal context (or downstream of a potential non-AUG start codon) may provide researchers with a testable assumption on the presence of additional regulatory signal influencing mRNA translation initiation rate and the start codon choice. *AUG_hairpin*, which has a convenient Web-interface with adjustable parameters, will make such an evaluation easy and efficient.

## Background

Translation of most eukaryotic mRNAs is likely to be initiated by a linear scanning mechanism [1], although some alternative mechanisms are also possible [2,3]. According to the scanning model, 40S ribosomal subunits can either initiate translation at the 5'-proximal AUG codon in a suboptimal context or miss it and initiate translation at downstream AUG(s). For mammalian mRNAs, the most important elements of AUG context are the adenine at position -3 and guanine at position +4 [1,4]. One might expect that mRNA should possess some features providing efficient translation, including the recognition of a genuine translation start site (TSS). However, the fraction of eukaryotic mRNAs with the start AUG codon in a suboptimal context is relatively large [5,6]. It is likely that at least some mRNAs with a suboptimal context of annotated start codon contain other signals providing additional information for efficient TSS recognition (*e.g*., [7]).

**Figure 1:**
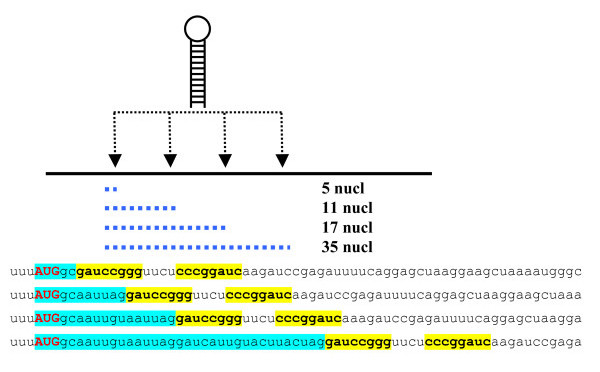
Description of experiment reported by Kozak ([8]): The hairpin (inverted repeats are marked with bold type and yellow boxes) was placed at the distances of 5, 11, 17, and 35 nucleotides from the CDS beginning (marked with blue boxes).

It has been reported earlier that stable hairpins located in certain positions downstream of AUG codon in a suboptimal context can increase translation initiation efficiency [8]. The hairpin was placed at the distances of 5, 11, 17, and 35 nucleotides downstream of the CDS beginning, and start codon recognition efficiencies were evaluated in *in vitro *experiment (Fig. 1). It was reported that the hairpin located at the distance of either 5 or 35 nucleotides did not increase the AUG recognition. However, the hairpin located at the distance of 17 or (to lesser extent) 11 nucleotides enhanced translation initiation considerably in a sequence-independent manner. It was hypothesized [8] that stable hairpins could temporarily stop the movement of 40S ribosomal subunits until the disruption of the secondary structure. The hairpin located at certain critical distance(s) with respect to start codon (at 17^th ^nucleotide in the experiment) could delay the 40S ribosomal subunit just in the position providing an efficient interaction between the met-tRNAi-located anticodon and the start AUG codon. It was also shown that such a downstream hairpin could increase translation from non-AUG start codons located in the optimal context [8,9].

This hypothetical mechanism has not been fully verified by *in vivo *experiments yet. However, it was often suggested that such secondary structures can be used to modulate translation efficiency of certain viral and cellular mRNAs with a suboptimal context of start AUG codons [10-14] or with a non-AUG start codons [15-17]. For example, the efficiency of TSS in a suboptimal context in Dengue virus mRNA is increased by a hairpin of moderate stability located 17 nucleotides downstream. The hairpin effect on the TSS recognition depended on its stability and position with respect to AUG [14]. It was also recently found that the stable hairpin located downstream of start AUG codon in Sindbis virus subgenomic 26S RNA provides efficient translation even though eIF2alpha is phosphorylated and translation of most cellular mRNAs is blocked [18]. The authors hypothesized that the hairpin can stall the ribosomes on the correct site to initiate translation, thus bypassing the requirement for a functional eIF2 and, thereby, specifically supporting translation of certain viral and (probably) cellular mRNAs. Notably, this function of a downstream hairpin is related to a general cellular translation control rather than a compensation of the "weakness" of the upstream start codon context. Thus, the information on the presence of potential compensatory hairpins may be useful for further experimental investigation of both general and specific mRNA translational properties.

Here, we describe the computational tool (*AUG_hairpin*) targeted at the prediction of secondary structure elements possibly compensating for suboptimal context of translational start codon. We also analyzed the structural features of human and mouse mRNAs and found significant correlation between the base pairing probabilities in positions 13–17 of CDS and the TSS context. This relationship supports the hypothesis on the functional significance of precisely located downstream hairpins for the TSS recognition in some cellular mRNAs.

## Implementation

According to the experimental data, the hairpins started either upstream or downstream of certain "critical" region of CDS did not compensate for the "weak" AUG context. In particular, continuous secondary structure started at 5^th ^nucleotide of coding sequence did not increase translation initiation efficiency despite it included the critical 12^th ^and 18^th ^positions (Fig. 1; [8]). Based on this observation, *AUG_hairpin *predicts the stem-loop structures, in which 5'-borders are located within the critical region (from 12^th ^to 18^th ^nucleotides by default). An appropriate stem-loop structure can also be a part of a more complex secondary structure started upstream of the critical region. We hypothesized that the 40S ribosomal subunit moving from the 5'-end of mRNA can pause consequently on each stable stem of a complex stem-loop structure waiting for its melting. In this case we assumed that an eligible hairpin has to be separated from upstream secondary structure elements by some impaired segment (*e.g*., loop) (for detailed description, see tutorial at the program www-sites).

5'-UTR and CDS nucleotide sequences have to be entered separately to the program through www-page (this provides the program with information on the start codon position). *AUG_hairpin *analyzes the mRNA segment compiled from 10 nucleotides long 5'-UTR portion located immediately upstream of the start AUG codon and 100 5'-proximal nucleotides of CDS. Algorithm consists of the following main steps: (1) Prediction of RNA optimal secondary structure for 5'UTR-CDS fragment. For this purpose the program *foldRNA *from Vienna RNA package v.1.4 [19] was implemented as subroutine. (2) Checking the occurrence of a perfect (or imperfect) stem located a certain distance downstream of start AUG codon (from 12^th ^to 18^th ^nucleotides by default; user can change this range from the CDS beginning till 30^th ^nucleotide). Conventionally, a stem is perfect when it does not contain any interrupting loops; an imperfect stem includes short mismatches (one-nucleotide bulges or 1+1 inner loops) which presumably do not interrupt stacking interactions. Program's output presents visualization of the optimal secondary structure and provides calculation of the predicted thermodynamic stability of the secondary structure and the helices (if any) started within the defined critical region. The program was written on C++ and runs in a Unix environment.

**Figure 2:**
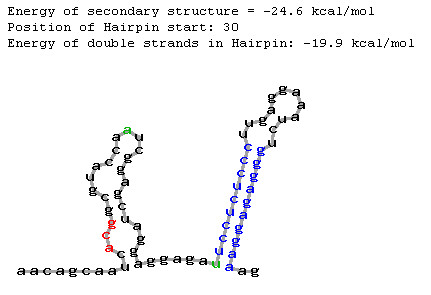
Predicted local secondary structure at the CDS beginning of *Agamous *mRNA (printscreen of *AUG_hairpin *output www-page; nucleotide sequence of 5'-UTR-CDS fragment was taken from [15]). Borders of the critical region (12^th ^and 30^th ^pos.) are marked with green colour; stem region of the eligible hairpin is marked with blue colour; start codon is marked with red colour.

**Figure 3:**
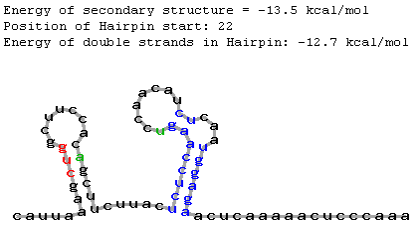
Predicted local secondary structure at the CDS beginning of *NsRpoT-C *mRNA (printscreen of *AUG_hairpin *output www-page; nucleotide sequence of 5'-UTR-CDS fragment was taken from AB084950 GenBank entry and [20]). Borders of the critical region (12^th ^and 30^th ^pos.) are marked with green colour; stem region of the eligible hairpin is marked with blue colour; start codon is marked with red colour.

It is likely that a downstream hairpin can increase translation even located outside the critical region defined by Kozak ([8]; from 12^th ^to 18^th ^nucleotides of CDS) although at a lower efficiency. It was experimentally found that the hairpin could influence translation even located at pos. 30 [10] or at pos. 27 of CDS [18]. Taking into account that the hairpin located at CDS position 35 did not increase an upstream AUG recognition in *in vitro *experiment [8], we limited the *AUG_hairpin *prediction interval by 30^th ^nucleotide of CDS. We analyzed two mRNA nucleotide sequences with annotated non-AUG translation start site taking them as examples. CDS of *Agamous *mRNA (*Arabidopsis thaliana*) starts from ACG codon [15] and CDS of *NsRpoT-C *mRNA (*Nicotiana sylvestris*) starts from CUG codon [20]. According to the *AUG_hairpin *prediction, there are stable hairpins located downstream of start codons in these mRNAs (*Agamous*: at pos. 30, E = -19.9 kcal/mol, Fig. 2; *NsRpoT-C*: at pos. 22, E = -12.7 kcal/mol, Fig. 3).

## Results and discussion

It was assumed that stable hairpins precisely located downstream of translation start sites (AUG or non-AUG) enhance translation initiation rate of some viral and cellular mRNAs [10-17]. Computational analysis of yeast mRNAs demonstrated the interrelationship between the base pairing probabilities at pos. 14–17 of CDS and the start codon context: mRNAs with a suboptimal TSS context were characterized by a more frequent presence of secondary structure at these positions [21]. In this work we tested the interdependency between the base pairing probabilities in the 5'-terminal CDS segment and start codon context in mammalian mRNAs. For this purpose human and mouse cDNA samples characterized by complete CDS and 5'-UTR size larger than 29 nucleotides were extracted from GenBank. The subsamples of cDNAs characterized by either optimal (purine at pos. -3 upstream the annotated TSS; 18750 human and 7700 mouse cDNAs) or suboptimal (pyrimidine at pos. -3 upstream the annotated TSS; 3469 human and 1233 mouse cDNAs) start codon context were used for further comparative analysis. The mRNA base pairing probabilities (BPP; [22]) were calculated using the Vienna RNA secondary structure package [19]. mRNA segments containing 5'-UTR (limited by 100 nucleotides in size) and the beginning of CDS (100 nucleotide in size; thus, total size of segments varied from 130 to 200 nucleotides depending on the 5'-UTR length) were used. Positional BPP values were calculated and compared between the samples of mRNAs with optimal and suboptimal start codon contexts. The difference in average BPP values at the CDS positions between these samples is shown in Table 1. One may see that mRNAs with a suboptimal TSS context are characterized by significantly higher average BPP values in pos. 13–17. We also calculated average BPP values at 5'-end CDS positions (from 6^th ^to 56^th ^nucleotides; average BPP = 0.615112 ± 0.0006 (human mRNAs with a suboptimal TSS context); 0.615775 ± 0.0006 (human mRNAs with optimal TSS context); 0.613197 ± 0.002 (mouse mRNAs with a suboptimal TSS context); 0.614936 ± 0.0009 (mouse mRNAs with optimal TSS context)). Thus, mRNAs with a suboptimal start codon context were not characterized by a higher average BPP value at the CDS beginning.

**Table 1:** The difference in average BPP values and G+C content in 6^th ^– 30^th ^CDS positions of human and mouse cDNAs characterized by optimal and suboptimal start codon contexts (purine (Pu) and pyrimidine (Py) in pos. -3 upstream AUG, respectively)†

CDS pos.	human		mouse	
	
	BPP^Py^-BPP^Pu^	GC^Py^-GC^Pu^,%	BPP^Py^-BPP^Pu^	GC^Py^-GC^Pu^,%
6	-0.014	-3.93	0.007	-1.16
7	0.005	-2.23	-0.016	-3.14
8	0.011	1.34	-0.002	0
9	0.006	-2.36	-0.006	-1.07
10	0.006	1.15	-0.014	-1.21
11	-0.008	2.14	-0.004	-1.47
12	-0.007	-3.79	0.005	2.46
13	0.015*	-0.41	-0.004	-0.38
14	0.012**	1.65	0.020*	4.47
15	0.015***	-1.99	0.018**	-1.08
16	0.014*	-0.93	0.036***	-1.48
17	0.012*	4.21	0.019*	7.53
18	-0.003	-3.23	-0.005	-5.46
19	0.004	0.59	0.010	1.31
20	0.005	0.68	-0.008	2.8
21	-0.025	-4.94	-0.012	-1.29
22	-0.005	-0.50	-0.013	-1.6
23	-0.018	3.79	-0.020	5.15
24	-0.020	-3.51	0.001	-3.21
25	0.007	-0.70	-0.013	-0.32
26	0.017*	2.14	-0.003	4.01
27	0.006	-3.66	-0.022	-4.23
28	-0.001	-0.03	-0.022	1.02
29	0.007	5.30	-0.022	5.21
30	-0.016	-3.68	-0.035	-5.06

† CDS positions where mean BPP values were significantly higher in mRNA samples with a suboptimal start codon context are marked (BPP^Py^-BPP^Pu ^values are bolded; the significance level (according to Mann-Whitney *U*-test) is shown by numbers: * p < 0.05; ** p < 0.01;*** p < 0.001)

It may be speculated that this difference results from a local deviation in G+C content (GC pairs make the major impact on the secondary structure stability) or from some codon-dependent periodic pattern [23,24]. However, average positional differences in G+C frequencies do not correlate with the difference in BPP values: it is unlikely that the observed dependency between the TSS context and base pairing probabilities reflects an unusual G+C distribution at the CDS beginning rather than the more frequent representation of the hairpin-containing mRNAs in a sample with a suboptimal TSS context (Table 1). These data demonstrate that precisely positioned hairpins may increase translation efficiency of some mammalian mRNAs *in vivo *and that the positions defined by Kozak ([8]; from 13^th ^to 17^th ^nucleotide of CDS) are most frequently (or efficiently) used for this purpose.

*AUG_hairpin *was used to analyze the stem-loop structures whose 5'-borders were located between the 6^th ^and 30^th ^nucleotides of CDS in human and mouse mRNA samples. Three parameters were taken into account: total energy of the stem-loop structure (E_tot_), energy of the stem (E_st_), and the stem size (L_st_, 5'-proximal perfect stems started within defined region were taken). It was found that mRNAs with a suboptimal start codon context were characterized by significantly more stable secondary structure elements started in positions 13, 14, 16, 17, 19 (Table 2). According to these results, the 5'-border of the critical region is also located at position 13 of CDS. We further selected subsamples of human and mouse mRNAs with stable stem-loop structures whose 5'-borders were located between the 13^th ^and 19^th ^nucleotides of CDS. It was found that 546 human mRNAs (16% of the sample with a suboptimal start codon context) were characterized by the presence of a stable stem-loop structure (E_tot _< -20 kcal/mol) in the defined region. Average energies of eligible stem-loop structures (E_tot_) were -34.9 kcal/mol and -33.2 kcal/mol for human mRNAs with a suboptimal and the optimal start codon contexts, respectively (the distributions of E_tot _values differ significantly according to Kolmogorov-Smirnov two-sample test, p < 0.01; the difference between mean E_tot _values was also significant according to the Mann-Whitney U-test, p < 0.00005). Similarly, 187 mouse mRNAs with a suboptimal start codon context were characterized with a highly stable hairpin (E_st _< -20 kcal/mol) in this region (E_tot _= -32.9 kcal/mol).

**Table 2:** The difference in characteristics of 5'-end proximal hairpins started in 6^th ^– 20^th ^CDS positions of human and mouse cDNAs characterized by optimal and suboptimal start codon contexts (purine (Pu) and pyrimidine (Py) in pos. -3 upstream AUG, respectively; E_tot_, energy of an eligible hairpin, kcal/mol; E_st_, energy of the hairpin stem region, kcal/mol; L_st_, size of the hairpin stem region, base pairs)†

CDS pos.	human			mouse		
	
	E_tot_^Py^-E_tot_^Pu^kcal/mol	E_st_^Py^-E_st_^Pu ^kcal/mol	L_st_^Py^-L_st_^Pu ^b.p.	E_tot_^Py^-E_tot_^Pu ^kcal/mol	E_st_^Py^-E_st_^Pu ^kcal/mol	L_st_^Py^-L_st_^Pu ^b.p.
6	0.45	0.21	-0.14	0.09	-0.16	0.08
7	0.40	0.38	-0.19	0.02	0.35	-0.26
8	-0.25	0.15	-0.22	1.04	-0.027	-0.16
9	1.79	0.11	-0.203	0.24	-0.53	0.02
10	1.89	0.01	0.13	-0.47	-0.29	0.11
11	0.79	0.21	-0.09	-0.87	-0.80	0.16
12	1.12	0.11	-0.02	0.86	1.29	-0.64
13	-1.28*	0.39	-0.13	-3.65*	-1.02*	0.19
14	-2.41*	-1.09**	0.10	1.12	1.65	-0.74
15	-0.87	-0.29	0.09	-1.84	0.54	-0.47
16	-1.03**	-0.53*	0.26*	3.42	-0.10	0.11
17	2.87	-1.15**	0.71*	-3.73*	-0.60*	0.46
18	-0.94	0.13	-0.24	3.08	-0.43	0.04
19	-2.99*	0.82	-0.48	1.88	0.75	0.10
20	-1.82	-0.58	0.06	-2.74	0.47	-0.32

† CDS positions where mean E_tot _and E_st _values were significantly lower (and L_st _values were significantly higher) in mRNA samples with a suboptimal start codon context are marked (E_tot_^Py ^– E_tot_^Pu^, E_st_^Py ^– E_st_^Pu^, and L_st_^Py ^– L_st_^Pu ^values are bolded; the significance level (according to Mann-Whitney *U*-test or Kolmogorov-Smirnov two-sample test) is shown by numbers: * p < 0.05; ** p < 0.01;*** p < 0.001)

It was reported that a correctly positioned hairpin even of a moderate stability (-8.2 kcal/mol) enhanced the recognition of upstream AUG codon in a suboptimal context [14]: thus, it may be assumed that at least some of these mammalian mRNAs possess higher translation initiation efficiency due to the presence of "compensatory" hairpins than it may be expected from the context of annotated start codon (lists of human and mouse mRNAs with some additional information on secondary structure characteristics are available as Additional file 1 and Additional file 2, respectively). It should be, however, noted that suboptimal context of start codon is not necessarily compensated by "compensatory hairpin": in many cases, contexts of translational start sites are likely to be evolutionary attenuated to decrease translation level of mRNAs encoding regulatory proteins (*e.g*., [5]).

## Conclusion

The presence of a potential secondary structure downstream of start AUG codon in a suboptimal context (or downstream of a potential non-AUG start codon) can provide researchers with a testable assumption on the additional regulatory element influencing translation initiation level. *AUG_hairpin *is based on an elegant hypothesis supported by the *in vitro *[8] and *in vivo *experimental data [10,12-14] as well as the results of computational analysis ([21]; Tables 1 and 2 in this manuscript).

It should be noted that the applied algorithm depends on the interpretation of available (rather limited) experimental data and the prediction accuracy may also be limited. Only few hairpin positions were tested in experiments. Secondary structure elements influencing translation start site recognition *in vivo *may have distinct characteristics (*e.g*., species-specific or tissue-specific). Currently it is also not possible to predict the interdependence of hairpin stability and its influence on start codon recognition as well as the influence of mRNA-protein and mRNA-ribosome interactions during translation initiation process on the mRNA secondary structure. Finally, the recognition of start codons in a suboptimal context can be modulated through other (currently poorly known) signals [7,25-28], and the absence of a "compensatory" hairpin does not necessarily mean that the TSS recognition is inefficient. However, despite these limitations, *AUG_hairpin *may be used to reveal potential "compensatory" hairpins in the case of discrepancy between the gene expression pattern and mRNA features (e.g., highly expressed gene is characterized by a suboptimal context of annotated translation start site, proteomic or phylogenetic data suggest the usage of non-AUG potential start codons [29], etc.).

## Availability and requirements

Project name: *AUG_hairpin*

Project home pages:





Any restrictions to use by non-academics: licence needed

## Abbreviations

UTR, untranslated region of mRNA; CDS, protein coding sequence; BPP, base pairing probability.

## Authors' contributions

AK and IT conceived of the study, carried out the computational analysis and drafted the manuscript. AP participated in the implementation of *AUG_hairpin *algorithm. NK and AS participated in the design and coordination of the study. DG prepared the www-site. All authors read and approved the final manuscript version.

## Supplementary Material

Additional file 1 List of human mRNAs with a suboptimal context of annotated start codon potentially containing "compensatory hairpins". This table provides a list of human mRNAs with a suboptimal context of annotated start codon containing stem-loop structures whose 5'-borders are located between the 13^th ^and 19^th ^nucleotides of CDS. The Table contains information on secondary structure (position of a hairpin's 5'-border, energy of the hairpin and stem(s) given separately) as well as an information from DE (DESCRIPTION) field of GenBank entries (which provides the names of corresponding genes). Click here for file

Additional file 2 List of mouse mRNAs with a suboptimal context of annotated start codon potentially containing "compensatory hairpins". This table provides a list of mouse mRNAs with a suboptimal context of annotated start codon containing stem-loop structures whose 5'-borders are located between the 13^th ^and 19^th ^nucleotides of CDS. The Table contains information on secondary structure (position of a hairpin's 5'-border, energy of the hairpin and stem(s) given separately) as well as an information from DE (DESCRIPTION) field of GenBank entries (which provides the names of corresponding genes). Click here for file
